# Physiological Function and Characterization of TRPCs in Neurons 

**DOI:** 10.3390/cells3020455

**Published:** 2014-05-21

**Authors:** Yuyang Sun, Pramod Sukumaran, Bidhan C. Bandyopadhyay, Brij B. Singh

**Affiliations:** 1 Department of Basic Science, School of Medicine and Health Sciences, University of North Dakota, Grand Forks, ND 58201, USA; 2 Calcium Signaling Laboratory, Research Service, Veterans Affairs Medical Center, Washington, DC 20422, USA

**Keywords:** Ca^2+^, TRPC channels, neuronal function, neurodegenerative diseases

## Abstract

Ca^2+^ entry is essential for regulating vital physiological functions in all neuronal cells. Although neurons are engaged in multiple modes of Ca^2+^ entry that regulates variety of neuronal functions, we will only discuss a subset of specialized Ca^2+^-permeable non-selective Transient Receptor Potential Canonical (TRPC) channels and summarize their physiological and pathological role in these excitable cells. Depletion of endoplasmic reticulum (ER) Ca^2+^ stores, due to G-protein coupled receptor activation, has been shown to activate TRPC channels in both excitable and non-excitable cells. While all seven members of TRPC channels are predominately expressed in neuronal cells, the ion channel properties, mode of activation, and their physiological responses are quite distinct. Moreover, many of these TRPC channels have also been suggested to be associated with neuronal development, proliferation and differentiation. In addition, TRPCs also regulate neurosecretion, long-term potentiation and synaptic plasticity. Similarly, perturbations in Ca^2+^ entry via the TRPC channels have been also suggested in a spectrum of neuropathological conditions. Hence, understanding the precise involvement of TRPCs in neuronal function and in neurodegenerative conditions would presumably unveil avenues for plausible therapeutic interventions for these devastating neuronal diseases.

## 1. Introduction

In neurons, Ca^2+^ is essential for a variety of physiological processes that regulate functions, such as gene transcription to neuronal growth, survival and even differentiation [1]. Ca^2+^ homeostasis is a tightly regulated process throughout the nervous system in order to maintain the tight Ca^2+^ balance between the intra- and extra-cellular fluids of the neuronal cells. Too much or too little Ca^2+^ can be deadly to these neurons, so the Ca^2+^ levels are carefully controlled in and outside of the cell. Such disturbances in Ca^2+^ homeostasis have been involved in neurodegenerative diseases, such as Parkinson’s, Alzheimer’s and Huntington’s [2,3], which is mainly due to the high dependence of Ca^2+^ signaling in various neuronal cells essential for their function [4]. Ca^2+^ mobilization in neuronal cells is tightly regulated by different Ca^2+^ channels and pumps in the plasma membrane and in the organelle membranes. An increase of intracellular Ca^2+^, specifically due to Ca^2+^ release from intracellular ER stores, as well as Ca^2+^ entry across the plasma membrane via various ion channels, including the endogenous store-operated Ca^2+^ entry (SOCE) channels, has gained much attention in recent years and will be the focus of this review. 

Although several mechanisms are known to control Ca^2+^ influx across the plasma membrane, Ca^2+^ influx could be more directly controlled either by store-depletion *per se* (through SOCE), or by the activation of the G-protein coupled receptors, or by the alterations in the membrane potential (through the activation of the voltage-gated Ca^2+^ channels). Since Ca^2+^ regulate such diverse processes, it is hard to pin-point a physiological function to one particular Ca^2+^ channel, and factors, such as amplitude, the amount of cytosolic Ca^2+^, the spatial distribution of individual Ca^2+^ channels and their modulators, may indeed be critical for regulating these diverse neuronal processes [5]. Although the significance of voltage-gated Ca^2+^ channels in neuronal cells is quite apparent, recent evidence have been gaining momentum to suggest an equally important role of SOCE channels. Ca^2+^ influx through SOCE mechanism is not only essential for the refilling of the ER Ca^2+^ stores, but is also critical for maintaining [Ca^2+^]_i_ that regulates neuronal functions, such as neurosecretion, sensation, long-term potentiation, synaptic plasticity, gene regulation, as well as neuronal growth and differentiation. SOCE channels also maintain a prolonged increase in cytosolic Ca^2+^ upon stimulation that is not only essential for the refilling of the ER Ca^2+^ stores, but can also activate many of the Ca^2+^-dependent processes that regulates neuronal functions. Two major families of proteins, Transient Receptor Potential Canonical (TRPC) and ORAI channels, have been identified in various cells including neurons, which have been shown to be critical for SOCE. Although the molecular components of SOCE have not been conclusively identified, especially in neuronal cells, ample evidence suggests the involvement of TRPCs in this process. Available data indicate that TRPC proteins initiate Ca^2+^ entry pathways and are essential in maintaining cytosolic, ER and mitochondrial Ca^2+^ levels. A number of biological functions have also been assigned to these TRPC proteins and are thus presented in this review [6,7,8,9]. 

## 2. TRPC Channels Properties and Their Mode of Activation

**Figure 1:**
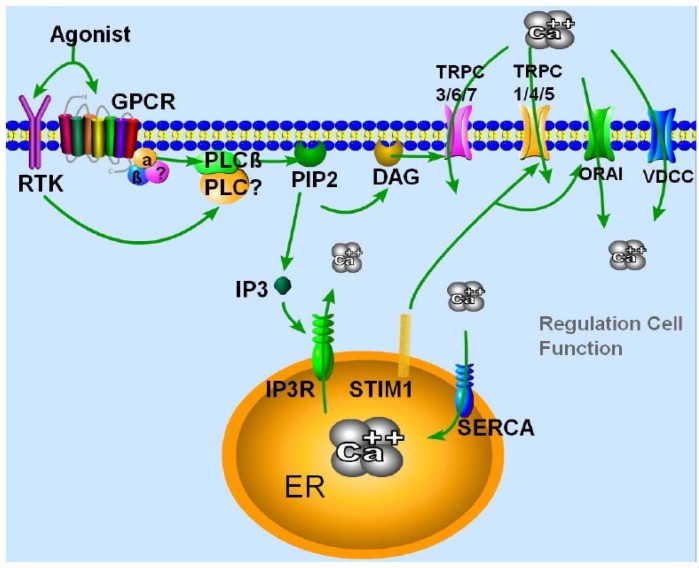
Activation and regulation of TRPC channels. Binding of an agonist to RTKs or GPCR, initiate a signaling cascade, causing PLC-mediated hydrolysis of phosphatidylinositol (4,5) bisphosphate (PIP_2_) to inositol (1,4,5)-triphosphate (IP_3_) and diacylglycerol (DAG). DAG could directly activate certain TRPC channels. IP_3_ binds to IP_3_R, a ligand-gated ion channel, which leads to the release of Ca^2+^ from the internal ER stores. Depletion of Ca^2+^ from the internal stores, in turn, allows STIM1 to aggregate, followed by the activation of the TRPC or ORAI channels in the plasma membrane, which allows Ca^2+^ to enter the cell that orchestrates cellular functions. It also could depolarize the membrane and activate the voltage-dependent Ca^2+^ channel (VDCC). SERCA pumps are shown to work concertedly to maintain steady-state levels of intracellular Ca^2+^.

Transient receptor potential (TRP) channels constitute a family of ion channels that are divided into TRPC (canonical/classical), TRPV (vanilloid) and TRPM (melastatin) sub-families. Importantly, all members of the TRP family are moderately conserved and share some homology among them, especially among each group member [10]. TRPC channels are an important Ca^2+^ influx pathway that is activated by store depletion *per se* and is present in most cell types. Generally, the binding of a hormone or a growth factor to its receptor is localized at the plasma membrane (PM). Activation of the G-protein (G_q/11_) leads to PIP_2_ hydrolysis, which generates IP_3_ and DAG [11] (Figure 1). IP_3_ binds to the IP_3_R (IP_3_ receptor) and initiates Ca^2+^ release from the ER stores, which empties the Ca^2+^ pool and initiates Ca^2+^ dissociation from the EF hand domain of the stromal interaction molecule-1 (STIM1) protein [12] (Figure 1). Importantly, STIM1 has been identified as the molecular link between ER Ca^2+^ store depletion and SOCE activation. Oligomerization of STIM1 occurs, followed by STIM1 translocation to the ER-PM junction, where it interacts with TRPCs and with some voltage-gated Ca^2+^ channels to modulate Ca^2+^ influx [13,14,15,16]. 

Although TRPC family contains 7 members (C1–C7) (Table 1) a unique property of these channels is that they all function as non-selective Ca^2+^ entry channels, with somewhat distinct mode of activation (Figure 1). However, based on their similarities with regard to the structure-function relationships, TRPC family can be further divided into two sub-groups. The first group consists of TRPC1/TRPC4/TRPC5 channels that can be regulated by receptor stimulation as well as by store-depletion, of which the latter requires the channel association with STIM1 and/or Orai1 proteins, thus are suggested as components of the SOCE channels [17,18]. On the other hand the second group comprises of TRPC3/TRPC6/TRPC7 that are activated by receptor stimulation [19]. TRPC2 is a pseudogene in humans, but has a role in rodent behavior and pheromone sensing [20]. It is however important to note that, although one can pharmacologically separate these channels *in vitro*, their activation in a physiological context is always linked to PLC mediated signaling following stimulation of membrane G-protein coupled receptors (GPCRs) or receptor tyrosine kinases (RTKs). Since TRPC proteins are capable of forming functional channels by heteromeric interactions [21], the receptor- or store-dependent activation thus outlines a common feature in channel activation [22]. Furthermore, regardless, as how these TRPC channels are regulated, they all contribute towards Ca^2+^ entry, which is fundamental for various neuronal functions.

Importantly, in neuronal cells, Ca^2+^ entry via the G-protein coupled mechanism has also been implicated in the shaping of action potentials, synaptic transmission and sensory transduction [23,24]. Additionally, changes in [Ca^2+^]_i_ are also known to regulate the motility of many cellular structures, including the axonal growth cones [25] and the dendritic filopodia of developing neurons [26]. Thus, it can be anticipated that TRPC channels may have a significant role in regulating these fundamental neuronal processes. 

**Table 1:** Expression of the TRPC channel and its properties in neuronal cells.

Subfamily	Cellular expression in Neurons	Ion channel properties	References
**TRPC1**	Brain, retina, peripheral axons and the mechanosensory terminals	Non selective, 16 pS current conductance, non-rectifying or mildly inward rectifying with a reverse potential of about +10 mV	[27,28,29,30]
**TRPC2**	Dendritic tips of the vomeronasal sensory neurons and spermatozoa (mouse)	Partially selective with a Pca/PNa ratio of 2.7, 42 pS current conductance, non-rectifying	[31,32]
**TRPC3**	Central nervous system (CNS)	Non selective, 60–66 pS current conductance, slightly dual (inward and outward) rectifying with a reverse potential of +5 mV	[33,34,35,36]
**TRPC4**	CNS, retina	Non selective, 30–42 pS current conductance, dual (inward and outward) rectifying with a reverse potential of about +10 mV	[37,38,39,40]
**TRPC5**	Brain, especially in fetal brain	Partially selective with a Pca/PNa ratio of 9, 47–66 pS current conductance, dual (inward and outward) rectifying as a homomer, outwardly rectifying when expressed with TRPC1 or TRPC4	[30,38,40,41,42,43]
**TRPC6**	Brain, retina	Partially selective with a Pca/PNa ratio of 5, 28–37 pS current conductance, dual (inward and outward) rectifying or inward rectifying	[33,35,44,45,46]
**TRPC7**	CNS (human); weak in CNS (mouse)	Partially selective with a Pca/PNa ratio of 5.9, 25–50 pS current conductance, slightly outward rectifying	[35,44,47,48]

## 3. Physiological Function of TRPCs in Neuronal Cells

Ca^2+^ plays a vital role in regulating various neuronal functions, like, neuronal survival, growth and differentiation. Ca^2+^ also acts as a ubiquitous secondary messenger to modulate neuronal functions and also plays an important role in the relay of information via membrane depolarization [4,49,50]. TRPCs are predominantly expressed in neuronal cells and, thus, could be prime contributors in regulating fundamental neuronal functions via regulating the Ca^2+^ flux in these neuronal cells [50]. Recent studies have shown an association of TRPC channels with neuronal development, synaptic functions and neuronal differentiation [51], and thus, they could be critical for neuronal function, as described below.

### 3.1. TRPC1

During neuronal development, every stage is involved in the compartmentalization of distinct neuron that utilizes Ca^2+^ and its downstream signaling molecules to perform varied complex processes that are essential for their development. Intracellular Ca^2+^ has diverse effects in shaping the axons and dendritic processes. Importantly, it has been shown that TRPC1 is highly expressed in embryonic CNS in mammals, compared with adults, indicating that TRPC1 could be involved in early development and the proliferation of neurons [52]. However, TRPC1 knockout mice are born healthy and survive in a control environment, but not in stressed conditions, without any moderate neuronal defects, suggesting that in the absence of TRPC1, perhaps other TRPC channels might compensate for its function [53]. In addition, there are slight discrepancies with regard to the involvement of TRPC channels in neuronal proliferation *vs*. regeneration, since TRPCs shows different levels of expression in neuronal populations that control cell proliferation, but not regeneration. Furthermore, the differences could be attributed to different neuronal populations that could have different expressions of TRPC isoforms, which could form heteromultimers and could therefore function differently in different tissues [43,53,54]. Stimulation of TRPC1 via different agonists could very well also have a different physiological response that could be attributed to the spatial temporal resolution of Ca^2+^ signaling in neurons. Interestingly, TRPC1, TRPC2 and TRPC4 have been shown to exhibit higher expression, whereas TRPC3 and TRPC6 expressions were decreased in neuronal stem cell (NSC) populations [55,56]. These results suggest that perhaps in NSC cells, TRPC1 could associate with TRPC4 rather than TRPC3 or TRPC5 and, thus, could bring about a different physiological function (probably regeneration). Interestingly, in most of the cases, the survival and proliferation induced by TRPC1 appears to be dependent on its Ca^2+^ entry; however, it remains to be seen if TRPC1 could potentially regulate other proteins, independent of its Ca^2+^ influx ability. TRPC1 has also been shown to be expressed in perisynaptic regions of the synapse and was physically associated with mGluR1 [57]. Furthermore, expression of a dominant-negative TRPC1-pore mutant (F561A) in cerebellar Purkinje neurons resulted in a 49% reduction of mGluR-evoked slow excitatory postsynaptic currents (EPSCs), whereas fast transmission mediated by AMPA-type glutamate receptors remained unaffected, indicating that mGluR1 receptor activation is essential for the gating of TRPC1. However, another report showed no localization of TRPC1 at the synapse, and no functional activity of TRPC1 was observed in these regions [43]. These discrepancies can again be due to several factors, and recent reports have shown that membrane targeting and regulation of TRPC1 was dependent on its association with lipid rafts and its functional interaction with STIM1 and caveolin1 [13]; however, this has not been confirmed in neuronal cells.

### 3.2. TRPC2

TRPC2 is a unique member of the TRPC sub-family, since its expression in mammals is lost and is considered a pseudogene in higher mammals [58,59]. Relatively little is known about its physiological significance and interaction with other Ca^2+^ regulating signaling molecules [60]. In rodents, the loss of TRPC2 expression results in the reduction in their ability to detect pheromones, leading to gender-specific behaviors [60,61]. TRPC2 is present in the vomeronasal organs (VNO), which are responsible for detecting water-soluble pheromones. Liman *et al.* showed for the first time the role of TRPC2 in vomeronasal sensory neurons, and ultrastructural analysis revealed that VNO sensory receptor cells express TRPC2 [32]. TRPC2 knockout mice had a phenotype in which the sensory response to pheromones in urine was abolished, and the male failed to recognize the difference between male and female [32,62,63]. TRPC2 knockout male mice started to show sexual behavior towards other male mice [63]. The female TRPC2 knockout mice started showing male characteristic behaviors, such as mounting, pelvic thrust and ultra-sonic vocalizations [60,64]. Although humans do not express TRPC2 channels and do not have similar VNO neurons (they are degenerated), they have a functional olfactory system, and it could be suggested that perhaps another TRPC isoform could decode olfactory cues and could be responsible for normal and abnormal social behavior in humans [65]. Thus, it would be interesting to probe this research further and establish if other TRPC isoforms are involved in abnormal human behaviors [66,67].

### 3.3. TRPC3

The expression of TRPC3 is highest in the brain, but the importance of TRPC3 in the nervous system is not completely understood. Initial studies by Li *et al*. [68] revealed a role for TRPC3 channels in BDNF signaling. Moreover, it was shown that in pontine neurons, Trk receptors and TRPC3 are expressed during the same developmental stages of the brain, and the BDNF-stimulated non-selective cationic current (I_BDNF_) was mediated by TRPC3. In another study using mouse cerebellar Purkinje cells, it was established that TRPC3 is required for the slow synaptic potentials and inward currents evoked by group I metabotropic receptor (mGluR1) synaptic signaling [60]. Similarly, in cerebellar granule neurons (CGNs), BDNF-induced elevation of Ca^2+^ contributes to axonal growth cone guidance [69]. The specific involvement of TRPC3 in BDNF-induced growth cone plasticity was further demonstrated by silencing endogenous TRPC3 or by expressing a dominant-negative TRPC3 that inhibited growth cone formation. Furthermore, in the pyramidal neurons of rat hippocampus an involvement of TRPC3 channels in BDNF-induced dendritic spine formation has also been reported [70]. In H19-7 rat hippocampal neuronal cells, TRPC3 along with TRPC1 was significantly increased under differentiating conditions, whereas the expression of TRPC4 and TRPC7 were decreased [52]. Similarly, overexpression of a dominant-negative form of TRPC3 or TRPC6 affects the nerve-growth-cone guidance by BDNF [71]. Thus, this reciprocal regulation of TRPC channel expression in these neurons suggests their developmentally important function. 

Ca^2+^ entry via the G-protein-coupled mechanism has also been implicated in the shaping of action potentials, synaptic transmission and sensory transduction [72,73,74]. In mice lacking TRPC3, but not TRPC1, TRPC4 or TRPC6, the mGluR1-mediated slow synaptic potentials were completely absent. This abnormality in the glutamate neurotransmission in the postsynaptic neurons has resulted in impaired walking behavior, thus establishing a fundamental role for TRPC3 channels in motor coordination [75,76,77]. In yet another study, to identify crucial gene products implicated in cerebral ataxia, a phenotype-driven dominant mutagenesis screen was performed, and an ataxic mouse mutant by the name of moonwalker (Mwk) mice was identified. These mice exhibited a gain-of-function mutation (T635A) and maintained sustained activation of TRPC3 channels, perhaps due to the lack of negative feedback regulation by PKCγ-mediated phosphorylation of TRPC3. As a result, diminished dendritic barbarization and progressive loss of Purkinje neurons were observed. Although these studies contradict one another, they still suggest that TRPC3 could have a pivotal role in Purkinje neurons, and future studies are needed to confirm the role of TRPC3 in ataxia. Another possibility could be that in both of these conditions, Ca^2+^ signaling is altered, and as a tight balance of Ca^2+^ signaling is essential for neuronal function, they might have different effects. Corroborating these results is another study with a TRPC3 knockout mouse model, where the TRPC3 promoter region was disrupted. These mouse exhibit atrophy and progressive paralysis, suggesting an obligatory role of TRPC3 channels in neuronal signaling, differentiation and development [78]. Importantly, TRPC3 has been shown to associate with SNARE complex proteins [79], indicating that TRPC3 could be important for neurosecretion. Furthermore, the receptor for activated C-kinase-1 (RACK1), a multifunctional scaffolding protein known to be a key regulator of various signaling cascades in the CNS, have been shown to interact with TRPC3 in cultured rat hippocampal neurons [80]. However, the functional implications of such an interaction are yet to be defined. Interestingly, TRPC3 in astrocyte contributes to the critical homeostatic functions necessary for the metabolic and trophic support of neurons in response to neuronal injury and inflammatory activation of glia [81]. 

### 3.4. TRPC4

TRPC4 has also been shown to be highly expressed in excitable cells and is involved in the response to neural injury, the regulation of neurite outgrowth and regulating neuronal exocytosis [82]. TRPC4 has also been shown to be co-expressed with TRPC5 in CA1 pyramidal neurons of the hippocampus, which play an important role in neuronal Ca^2+^ homeostasis [71]. In gastrointestinal pacemaker cells and in mouse visceral smooth muscle cells, TRPC4 is also important for the control of muscarinic stimulation [83]. ATP induces an increased expression of TRPC4, which requires cAMP response element-binding protein (CREB) phosphorylation [84]. Studies by Huang *et al*. 2007 have further shown that TRPC4 expression is restricted to granule and their precursor cell in rat cerebellum, suggesting that TRPC4 is perhaps important for proper granule cell development; however, its function *in vivo* is still not well defined. TRPC4 is also shown to have a role in acute and delayed neuronal injury in focal cerebral ischemia [85], suggesting that TRPC4 could be a viable target, and its inhibition could protect against cerebral ischemia.

### 3.5. TRPC5

TRPC5 also plays an important role in the CNS and is important in neuronal function. TRPC5 is mainly involved in the regulation of hippocampal neurite length and growth cone morphology in young rat hippocampal neurons [60,86]. TRPC5 also regulates neurite outgrowth [87]. In neurons, BDNF triggers Ca^2+^ release from the internal stores via activation of the PLC-γ-IP_3_ pathway, which, in turn, activates the TRPC channel and allows the enhanced elevation of Ca^2+^, which is required to trigger the attractive turning of the neurons. In cultured hippocampal neurons, the expression of DN-TRPC5 elevates neurite extension, whereas overexpression of wild-type TRPC5 inhibits neurite growth [86]. Hence, TRPC5-containing channels may allow a larger Ca^2+^ influx than TRPC3/TRPC6 channels, leading to neurite growth inhibition [36,86], whereas a modest level of Ca^2+^ influx through TRPC3/TRPC6 channels is sufficient to trigger growth-cone tuning. Thus, by allowing different patterns of Ca^2+^ influx, diverse TRPC channels may thus carry out distinct functions at the growth cone. Axon formation via CAMPKK activation of CAMPK was suppressed with TRPC5 knockdown [88]. Studies by Wu G. *et al*. identified TRPC5 protein on the cell bodies of CGNs and demonstrated TRPC5 channels as a critical modulator of this PLC-pathway-mediated neurite outgrowth [89]. C5 knockout mice were shown to exhibit diminished innate fear levels in response to innately aversive stimuli. It has been reasoned that the lack of TRPC5 channel potentiation by Group I mGluRs and/or CCK2 receptors and the subsequent lack of membrane depolarization prevents the transmission of information to output neurons of the innate fear circuitry, thus resulting in the above-mentioned fear-related behavior [90]. Yan *et al*. in 2009 showed that TRPC5 channels have a role in generating Ca^2+^-activated slow after depolarization (sADP) currents signaled by muscarinic receptors [91].

### 3.6. TRPC6

TRPC6 is widely expressed in the cardiac neurons [92], in retinal ganglion cells [93], in the neurons of olfactory epithelium [94] and in parts of the brain, such as cortex, substantia nigra, hippocampus and cerebellum [95,96]. TRPC6 transgenic mice (that overexpress a mutant TRPC6) showed enhancement in spine formation and spatial learning and memory in Morris water maze [97,98]. Activation of Neurontin receptors by substance-P in the noradrenergic A7 neurons has been shown to bring about a TRPC6-specific non-selective cationic conductance, thus providing evidence for the involvement of TRPC6 channels in nociception [99]. TRPC6 in neurons has been shown to co-operate with other TRP channels, in a heteromeric assembly, to regulate critical neuronal functions. The significance of heteromeric TRPC6 channels in brain development is underscored by the finding that, in embryonic rat brain, TRPC6 physically associates with TRPC1, TRPC4 and TRPC5, respectively [43]. In dorsal root ganglion (DRG) neurons, TRPC6 co-operates with TRPC1 and TRPV4 to regulate nociception [53]. TRPC6, in conjunction with TRPC3, is involved in growth-cone guidance [36]. In rat cerebellar granule neurons, BDNF-induced activation of TrkB receptors resulted in a Ca^2+^-dependent growth-cone turning. BDNF-induced Ca^2+^ influx was shown to be mediated via TRPC3/TRPC6 channels. In the primary mid-brain neurons of rat, TRPC5 and TRPC6 co-localized with PDGF-βR and were found to regulate TRPC6 silencing, also abolishing the effect of BDNF on spine formation. This effect of TRPC6 channels on dendritic spine formation was largely mediated by the activation of the CaMKIV-CREB pathway [98].

### 3.7. TRPC7

TRPC7 is expressed in the nervous system (dorsal root ganglion cells), keratinocytes, uterine myometrium and in leukemia cells [60]. During pregnancy, in the uterus [100], and during development in dorsal root ganglion cells, the TRPC7 expression changes [101]. TRPC7 in rodents via a calcineurin-dependent pathway mediates the angiotensin II-induced myocardial apoptosis [102]. PGE2-induced apoptosis in K562 human leukemia cells is regulated by TRPC7 [103]. Ablating the expression of both TRPC6 and TRPC7 eliminated the intrinsic light response of the M1-subtype of melanopsin-expressing, intrinsically-photosensitive retinal ganglion cells [104]. 

## 4. TRPC Channels in Neurodegenerative Diseases

Changes in the intracellular Ca^2+^ concentration stimulate a number of intracellular events and could either trigger or inhibit cell death process, leading to neuronal injury. Furthermore, neuronal injury is a consequence of both the increase and decrease of cytosolic Ca^2+^ [5,105], and disturbances in Ca^2+^ homeostasis have been implicated in neurodegenerative diseases, such as, PD, AD and HD [106,107,108,109,110,111]. Although it is not surprising that disturbances in Ca^2+^ signaling pathways underlie neuronal loss, the cellular mechanism(s) underlying neurodegeneration remains to be elucidated [112]. However, as many factors involved in neuronal function are dependent on Ca^2+^ signaling, it could be anticipated that the loss of these critical functions could make these neurons vulnerable [5,105]. 

Several factors that contribute towards neuronal injury, including the generation of free radicals, the impairment of mitochondrial function, ER stress and apoptosis, have been proposed to be regulated by alterations in cytosolic Ca^2+^. Additionally, some of these factors have also been shown to be involved in the activation of TRPC channels and their regulator, STIM1 [113,114,115,116]. TRPC1 has been shown to be activated by STIM1, and recently, STIM1 has been shown to be activated by ROS [117,118]. Furthermore, as neurons are heavily dependent on mitochondrial function and have a high requirement for ATP, they could be of high importance, and Ca^2+^ entry has been shown to be vital for ATP synthesis. Similarly, as a byproduct of increased mitochondrial function, the generation of reactive oxygen species (ROS) is increased, which may further advance these neurons towards degeneration. Furthermore, postsynaptic scaffolding proteins have been shown to be associated with TPRC channels that are associated with the inhibition of intracellular Ca^2+^ overload-mediated ROS generation by TRPC channels in neurotoxin-induced neuronal injury [119]. Although at present, the consequence of ROS-mediated activation of STIM1-TRPC1 with regard to neuronal degeneration is not yet identified, it could be plausible that this could be one of the mechanisms that could lead to neuronal loss. Moreover, increased cytosolic Ca^2+^ leads to inappropriate activation of Ca^2+^-dependent processes, which stay inactive at low Ca^2+^ levels, causing metabolic derangements, leading to neuronal death [5,105,112]. Additionally, this could also lead to the loss of mitochondrial membrane potential, depletion of ATP and increased free radical production, creating oxidative stress within the cell [120]. The free radicals thus released from the mitochondria disturb the intracellular Ca^2+^ homeostasis, possibly by compromising the function of the Ca^2+^ signaling components of the ER and PM [121]. With regard to ER store-depletion, the cell has its own way of replenishing the stores through the activation of SOCE channels. Additionally, neurotoxins that mimic the degeneration of dopaminergic neurons drastically decrease TRPC1 expression [122]. As a result, the ER fails to maintain the normal Ca^2+^ levels required for its proper functioning, eventually leading to ER stress [123,124]. The mitochondrion, on the other hand, takes up the excess Ca^2+^ present in the cytoplasm (released from the ER) and loses its membrane potential due to Ca^2+^ overload. This, in turn, exacerbates the free radical production, thus creating a vicious cycle of oxidative stress, which initiates an intrinsic apoptotic cascade. Our findings also suggest that the overexpression of TRPC1 combats some of the negative effects of MPP+-induced oxidative stress and mitochondrial dysfunction, thus providing the cells an opportunity to recover from the stressed state.

Another mechanism that could also affect neuronal viability is ER stress. ER Ca^2+^ is essential for the proper folding and synthesis of neuronal proteins, and a decrease in ER Ca^2+^ can induce ER stress, which can activate cell death cascades [125]. In addition, in all of these neurodegenerative processes, abnormal protein folding is the key, suggesting that controlled Ca^2+^ influx from external media is critical for neuronal functioning and survival. Since TRPCs are essential for replenishing and for maintaining ER Ca^2+^, the chronic depletion of ER Ca^2+^, as would occur in the absence of TRPC function, could influence ER-dependent processes, such as protein folding and trafficking, the ER stress response and apoptosis. Accumulating evidence over the last decade has shown a strong link between the pathogenesis of AD and impaired Ca^2+^ homeostasis [112]. Furthermore, TRPC1 has been shown to be essential for Huntington disease [126], and mutations in TRPC1 will affect the risk of late-onset AD [127]. However, the mechanisms of it need further clarification. Interestingly, presenilins are also suggested to function as ER Ca^2+^ leak channels [128] and have been shown to be mutated in some AD cases. Importantly, mutant PS1 and PS2 transgenic mice, as well as neurons expressing presenilin mutations have been shown to have larger Ca^2+^ stores and an increased dynamic flow of Ca^2+^ into the cytosol [129,130]. The proposed mechanism for this effect is reasoned to be due to the disruption of the Ca^2+^ leak function of mutant presenilin, creating an imbalance between Ca^2+^ leak and Ca^2+^ reuptake. Contrary to this ‘Ca^2+^ overload’ hypothesis, there is strong evidence showing no increase in Ca^2+^ stores, but rather, Ca^2+^ stores in PS1 and PS2 mutant phenotypes are reduced [131,132,133]. It has also been shown that FAD PS1 and PS2 mutants interact with IP_3_R, rendering it sensitive to lower IP_3_ concentrations, thus affecting the receptor’s gating and resulting in exaggerated [Ca^2+^]_cyt_ [133]. These discrepancies could be attributed to the type of mutations on the presenilin and the cell type used, which would dictate the eventual phenotypic disparity. However, recently, it has been identified that PS2 and ORAI2 prevented the overload of ER Ca^2+^, which may regulate PIP_2_ levels to control Ca^2+^ extrusion via a feedback mechanism [134].

Another possibility is that SOCE could provide a mild conditioning stress that activates adaptive cellular stress responses, resulting in the upregulation of genes encoding neuroprotective proteins, such as neurotrophic factors, protein chaperones and antioxidant enzymes [135]. Consistent with this mechanism, transient Ca^2+^ influx is known to be required for the neurotrophic effects of glutamate receptor activation, and TRPC1 channels have been shown to mediate the actions of the brain-derived neurotrophic factor (BDNF) at some synapses [136]. Ca^2+^ may also mediate the cell survival-promoting function of glial cell line-derived neurotrophic factor (GDNF) in substantia nigra dopaminergic neurons [137]. The involvement of neurotrophic factor signaling upstream or downstream of TRPC1 channel activation may be part of a previously unknown adaptive neuronal stress response pathway [138]. Changes in the content of Ca^2+^ stores directly affect SOCE, and it has been shown that SOCE was abrogated with most of the presenilin mutations. Consistent with this, the knockdown of PS1 showed a marked potentiation of SOCE, suggesting that PS1 in general negatively regulates SOCE, and their mutations create a gain-of-function phenotype, which would further augment the inhibition. Although, at present, the direct identification of any TRPC channels is missing, a recent study showed that mouse embryonic fibroblast lacking presenilins had increased levels of STIM1 and decreased levels of STIM2 expression with a marked potentiation of SOCE. Consist with this, STIM2 has been shown to mediate neuronal SOCE, which continuously activated the Ca^2+^/calmodulin-dependent protein kinase II, which was decreased in mouse models of AD [139]. However, in another report, expression of FAD mutants in these cells attenuated SOCE without altering the STIM expression [140]. PS2 has been shown to influence TRPC6-mediated Ca^2+^ entry [131]. Coexpression of PS2 or PS2 mutants with TRPC6 completely abrogates agonist-induced Ca^2+^ entry through TRPC6; however, these studies were performed in non-neuronal cells, and it is not clear if a similar mechanism occurs in neurons. In spite of the mounting evidence for the role of Ca^2+^ in general and SOCE specifically in the pathogenicity of AD, mechanistic insight in this regard is lacking. Thus, in this context, the question of how the PS1 mutants regulate the TRPC channels needs further clarification.

## 5. Conclusions

In conclusion, we have discussed the involvement of TRPC channels in neuronal function; however, as seven TRPCs are expressed in neuronal cells, it is likely that they all could compensate for the loss of a particular TRPC channel. Nevertheless, accumulating evidence indicates that TRPC channels mediate physiological function in neuronal cells. Therefore, establishing their impact on pathophysiology and neuronal diseases is critical. Furthermore, increasing knowledge regarding the link between TRPC channels and various neuronal diseases should also lead to a fruitful development of innovative drugs or therapies to combat these deliberating diseases, as currently, no viable therapies are available for these patients. In addition, as some of these TRPC have redundant functions, it would be essential to develop KO mice with various TRPC deletions, as that could provide a much better scenario and establish their function in particular diseases. Furthermore, for clinical and translation aspect new areas of research such as: does SNPs, epigenetics modulations, and mutations regulate TRPCs expression and function; how predictive/useful will the animal models be in order to test drugs that can modulate TRPC channel function; can TRPC modulators identified using animal models, be safe and effective in combating neuronal diseases are still needed.

## Abbreviations

TRPC: transient receptor potential canonical cation channels
STIM: stromal interaction molecule
PKC: protein kinase C
ATP: adenosine triphosphate
PLC: phospholipase C
IP_3_R: inositol trisphosphate receptor
SERCA: sarco/endoplasmic reticulum Ca^2+^-ATPase
ER: endoplasmic reticulum
PM: plasma membrane
DAG: diacylglycerol
ORAI: calcium release-activated calcium channel protein
SOCE: store-operated calcium entry
GPCR: G-protein coupled receptor
BDNF: brain-derived neurotrophic factor
CNS: central nervous system
PD: Parkinson’s disease
AD: Alzheimer’s disease
HD: Huntington’s disease
RTKs: Receptor Tyrosine Kinases
Pca/PNa: Possible ratio.
